# Using Cellulose Nanocrystal as Adjuvant to Improve the Dispersion Ability of Multilayer Graphene in Aqueous Suspension

**DOI:** 10.3389/fbioe.2021.638744

**Published:** 2021-02-10

**Authors:** Haiqiao Zhang, Yan Wu, Feng Yang, Huiling Dong, Yuqing Bian, Huanliang Jia, Xuqin Xie, Jilei Zhang

**Affiliations:** ^1^College of Furnishings and Industrial Design, Nanjing Forestry University, Nanjing, China; ^2^Co-Innovation Center of Efficient Processing and Utilization of Forest Resources, Nanjing Forestry University, Nanjing, China; ^3^Fashion Accessory Art and Engineering College, Beijing Institute of Fashion Technology, Beijing, China; ^4^Dehua Tubao New Decoration Material Co., Ltd., Huzhou, China; ^5^Department of Sustainable Bioproducts, Mississippi State University, Mississippi State, MS, United States

**Keywords:** dispersion, ball-milling, multilayer graphene, cellulose nanocrystal, sedimentation analysis

## Abstract

Cellulose nanocrystal (CNC) has been applied in various fields due to its nano-structure, high aspect ratio, specific surface area and modulus, and abundance of hydroxy groups. In this work, CNC suspensions with different concentrations (0.4, 0.6, and 0.8%) were used as the adjuvant to improve the dispersion ability of multilayer graphene (MLG) in aqueous suspension, which is easy to be aggregated by van der Waals force between layers. In addition, N-methyl-2-pyrrolidone, ethanol, and ultrapure water were used as control groups. Zeta potential analysis and Fourier transform infrared spectroscopy showed that the stability of MLG/CNC has met the requirement, and the combination of CNC and MLG was stable in aqueous suspension. Results from transmission electron microscopy, Fourier transform infrared spectroscopy, and absorbance showed that MLG had a better dispersion performance in CNC suspensions, compared to the other solutions. Raman spectrum analysis showed that the mixtures of 1.0 wt% MLG with 0.4% CNC had the least defects and fewer layers of MLG. In addition, it is found that CNC suspension with 0.8% concentration showed the highest ability to disperse 1.0 wt% MLG with the most stable performance in suspension. Overall, this work proved the potential application of CNC as adjuvant in the field of graphene nanomaterials.

## Introduction

Graphene nanomaterials have been widely used as electrode materials for supercapacitors (Ouyang et al., 2013) and in composites with enhanced electrical (Gao et al., 2013), mechanical (Zhang et al., 2012), and thermal (Shahil and Balandin, 2012) properties because of their excellent properties. However, the large specific surface area and van der Waals forces (Atif and Inam, 2016) between layers induce graphene nanomaterial to agglomerate (Li et al., 2018) when dispersed into other materials as property enhancement agents, thereby causing lower performance of these nanomaterials as property-enhancement agents. Overcoming the agglomeration issue to fully take advantage of the superior properties of these nanomaterials becomes an important research topic.

The current method of dispersing graphene nanomaterial into a matrix is to disperse it in an aqueous surfactant solution first (Lotya et al., 2010). This is because the surfactant molecules attach onto the graphene nanomaterial surface through electrostatic repulsion or intermolecular forces, resulting in the uniform and high concentration dispersion of graphene nanomaterial in the solution. Surfactant aqueous solutions, such as ionic and non-ionic surfactants (Guardia et al., 2011), polymers (Wajid et al., 2012), organic salts (Du et al., 2013), and aromatic molecules (Geng et al., 2010; Zhang et al., 2010; Das et al., 2012), can be used both in organic and aqueous media to slow down reactions, maintain chemical balance, change surface tension, and prevent light and thermal decomposition or oxidative decomposition from happening. The direct exfoliation technique and stabilization of graphite in aromatic solvents or ionic liquids (Wang et al., 2010) suffer from low throughput and are a threat to human health and the environment (Yang et al., 2020). The aqueous surfactant solution has the advantages of low cost and being easily operated, but the concentration of the obtained graphene nanomaterial dispersion is relatively low. Some physical processes have also been applied to improve the dispersion of graphene nanomaterial in the solution mentioned above, like shearing (Knieke et al., 2010; Paton et al., 2014; Dong et al., 2018), microwave (Chiu et al., 2012), ball-milling (Zhao et al., 2010; León et al., 2014), and ultrasonic treatment (Nuvoli et al., 2011; Notley, 2012).

Herein, we demonstrated an approach to disperse multilayer graphene (MLG) in water with the cellulose nanocrystal (CNC) as adjuvant (Wu et al., 2019b). Cellulose, one of the most abundant, natural, and renewable biopolymers in the world, is widely present in biomasses like woods (Wu et al., 2019c, 2020a,b), corn stover (Zhao et al., 2020), and tunicate (Zhao et al., 2015), and can be metabolized by specific bacteria.

Cellulose is a long-chain polymer with repeated units of D-glucose, a simpler sugar form. The CNC is a nanomaterial that can be obtained by continuously peeling cellulose. The theoretical Young’s modulus of CNC along its chain axis is 167.5 GPa (Wajid et al., 2012) and tensile strength is 7.5–7.7 GPa (Gray et al., 2018). Furthermore, the features of inherent renewability, biodegradability, and biocompatibility make it an eco-friendly green material. In general, extraction from natural fibers through sulfuric acid hydrolysis is usually used to prepare CNC (Wu et al., 2019b). The negative charged sulfate groups in the CNC surface prepared by this method make them repel each other and result in good stability (Jiang and Hsieh, 2013).

**FIGURE 1:**
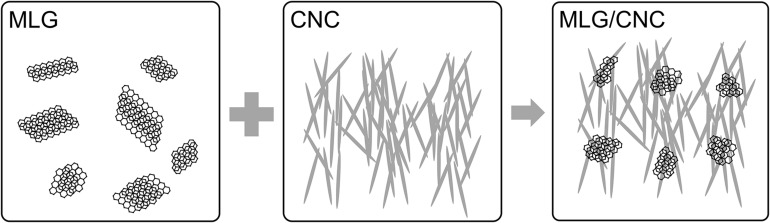
Schematic of the mechanism for dispersion of MLG in CNC aqueous suspension.

Herein, we demonstrated an approach to disperse MLG in water with the sulfuric acid hydrolysis CNC as adjuvant. The mechanism of CNC-assisted MLG dispersion in aqueous suspension is shown in Figure 1. The mechanism can be explained as the CNC aqueous suspension is sufficiently stable, and the MLG is attached to the “3D net” formed by CNC. In this study, MLG was ball-milled first. The ball-milling technology utilized the shear force generated by ball collision to offset the van der Waals force between the MLG layers (Guo and Chen, 2014), which can be physically peeled to reduce the size of MLG. Subsequently, through comparative experiments, the influence of CNC suspensions with different concentrations as adjuvant on the dispersion ability of MLG in water was evaluated.

## Materials and Methods

### Materials

The microcrystalline cellulose (MCC), N-methyl-2-pyrrolidone (NMP, AR), and ethanol absolute (AR) were purchased from Sinopharm Chemical Reagent Co., Ltd. (Shanghai, China). Sulfuric acid (AR, 98%) was obtained from Nanjing Chemical Reagents Co. (Nanjing, China). The ultrapure water was prepared by Plus-E3-10th (EPED, Nanjing, China). The MLG, a bio-MLG material (Yan et al., 2018), was provided by the Department of Sustainable Bioproducts, Mississippi State University.

### Sample Preparation

#### Ball-Milling MLG

For ball-milling, some grinding balls with a diameter of 3 mm and a certain amount of MLG (the weight ratio of ball to powder was 5:1) were placed in a 25-ml tank of a Tehtnica Millmix 20 homogenizer (DOMEL, Železniki, Slovenia), and the grinding time (10, 30, and 60 min) and oscillation frequency (20 Hz) were selected before running the machine. After ball-milling, the particle size of ground MLG was measured by a BT-90 nanoparticle size distribution analyzer (Bettersize, Dandong, China).

#### CNC Suspensions Preparation

The CNC aqueous suspension was prepared through the sulfuric acid hydrolysis of MCC (Cranston and Gray, 2006). The procedure started with hydrolysis at 45°C for 60 min, followed by acid removal, dialysis, and ultrasonic treatment.

#### MLG/CNC and Control Group Preparation

The MLG/CNC and other control mixtures (mixture with NMP, ethanol, and ultrapure water) were prepared according to Table 1. The MLG/CNC mixing procedure started with adding ball-milled MLG (the amount added are 0.5 and 1.0 wt%) to CNC aqueous suspension (0.4, 0.6, and 0.8%), followed by performing the ultrasonic treatment for 10 min (Q700, QSonica, Newtown, CT, United States). The control groups also underwent magnetic stirring and ultrasonic treatment with the same power and time. The MLG/CNC and control groups were loaded into the sample bottles and kept for 135 days for sedimentation analysis.

**TABLE 1:** The concentration of adjuvant or dispersant and the addition ratio of MLG in each sample.

Sample	Adjuvant or Dispersant (%)		MLG (wt%)
1	CNC	0.4	0.5
2		0.4	1.0
3		0.6	0.5
4		0.6	1.0
5		0.8	0.5
6		0.8	1.0
7	NMP	100	0.5
8		100	1.0
9	Ethanol	100	0.5
10		100	1.0
11	Ultrapure water	–	0.5
12		–	1.0

### Performance Testing

For particle size test, 0.01 g of ball-milled MLG was mixed with 20 ml of ultrapure water in a beaker, and the beaker was placed in a Q700 tip ultrasonicator for 2 min. After sonication, a total of 4 ml of suspension was loaded into a quartz cuvette for the test. This test used the automatic mode of nanoparticle size distribution analyzer. The zeta potential values of CNC aqueous suspension, MLG/CNC, MLG/NMP, MLG/ethanol, and MLG/ultrapure water were determined employing the Zetasizer Nano-ZS ZEN3600 (Malvern, Worcestershire, United Kingdom) provided with a 4-mW He–Ne (633 nm) laser. Except for 0.4, 0.6, and 0.8% CNC, all samples were diluted to 0.01 wt%, of which MLG/CNC samples 11–12 were diluted with the original dispersant. Samples 7–10 were diluted by ultrapure water due to their particularity (NMP will damage the inner wall of the sample cell, and the test results of alcohol do not meet the test quality requirement). The pH values of CNC aqueous suspension and MLG/CNC were around 3.0–3.5, and samples 7–12 were around 7.0. Using a syringe to transfer about 1 ml of the sample to the DTS1060 sample cell, after expelling the air bubbles, seal it with the stopper and perform the test. The sedimentation analysis of the MLG/CNC stored for 135 days was performed using the visual observation and microscopic characterization technique. The morphologies of CNC were observed by atomic force microscopy (Dimension Edge, Bruker, Germany) with a scan size of 1 μm × 1 μm. The transmission electron microscopy (TEM, JEM-1400, JEOL, Japan) was performed on the morphologies of MLG/CNC. As to TEM analysis, the diluted MLG/CNC was cast on copper grids for observation after drying. The absorbance analysis of the sample was performed using a U-3900 UV/vis/NIR spectrophotometer (Hitachi, Japan) at 660 nm. Use a “positioner” to get a certain height sample in the sample bottle, diluted at certain times to determine the absorbance. The sample was placed in the test cell, and the corresponding adjuvant was used as the reference. The Fourier transform infrared spectroscopy (FTIR) of the dried sample was measured using a VERTEX 80V FTIR spectrometer (Bruker, Germany) with a wavenumber range of 4,000–400 cm^–1^. The sample was dried in an oven and ground into powder and passed through a 200-mesh screen. Then, the powder was collected and mixed with potassium bromide (KBr) to make a transparent disk for testing. The dried MLG/CNC powder was put on a glass slide, and Raman spectra were carried out using DXR532 (Thermo Fisher Scientific, Asheville, NC, United States) with an excitation laser with λ = 532 nm.

## Results and Discussion

### Size Analysis of Ball-Milled MLG

**FIGURE 2:**
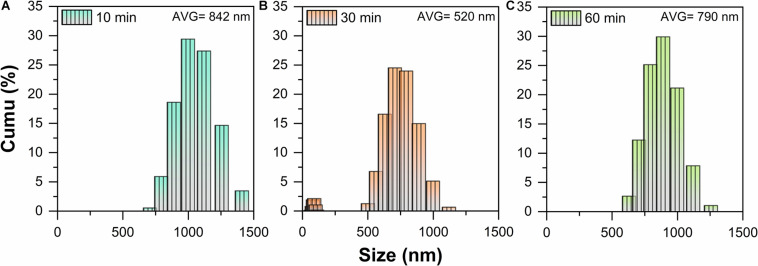
The particle size distribution of MLG at different ball-milling times **(A)** 10 min, **(B)** 30 min, and **(C)** 60 min.

Under the abovementioned conditions, the particle size distribution of ball-milled MLG at different treatment times is shown in Figure 2. The MLG size before ball-milling has reached the micron level, up to 2 μm or more (measured by Adobe Photoshop CC 2015). After grinding for 10 min, the average particle size of MLG was 842 nm, the average particle size of MLG was 520 nm after grinding for 30 min, and the average particle was 790 nm after grinding for 60 min.

This result indicated that the 30-min treatment time has the smallest particle size. The reason may be insufficient grinding after 10 min of treatment. For 60 min of ball-milling, the ball mill machine has generated lots of heat, causing the separated MLG to agglomerate again.

It can be known from Figure 2B that MLG has two size ranges. On the one hand, the reason was that MLG was wide and thin and not a sphere, and the nanoparticle size distribution analyzer detects its thickness and width, so there were two size ranges. On the other hand, the size of the MLG was not uniform, inducing the results concentrated in two size ranges. In view of this result, the MLG used later in this study was prepared with an oscillation frequency of 20 Hz and a ball-milling time of 30 min.

### Zeta Potential Analysis

**FIGURE 3:**
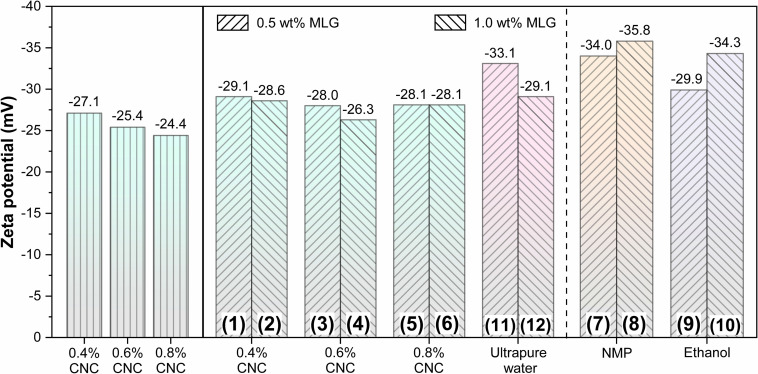
Zeta potential of CNC, MLG/CNC, and control groups.

Zeta potential was usually used to evaluate the stability of the colloid (Zhong et al., 2012); a zeta potential value greater than 30 mV or less than -30 mV was considered solely stabilized (Cao et al., 2016). The zeta potential results of CNC, MLG/CNC, and control groups are shown in Figure 3. The zeta potential values of 0.4, 0.6, and 0.8% CNC aqueous suspension were −27.1, −25.4, and −24.4 mV, respectively. According to the results, the zeta potential of each group of samples after dispersion was around −30 mV, indicating that samples 1–11 can basically meet the requirements of stability. The surface of CNC was negatively charged due to the preparation process of sulfuric acid oxidation (Chatterjee et al., 2020). This result showed that as the concentration of CNC aqueous suspension increased, its zeta potential decreased; that is, the stability was worse. The reason was that with the concentration increased, the distance between colloidal particles was reduced, and the electrostatic double layer has been compressed, inducing the decrease in zeta potential (Hsiung et al., 2016). The zeta potential values of the MLG/CNC were higher than those of 0.4, 0.6, and 0.8% CNC aqueous suspensions. This was because MLG also showed a negative charge when dispersed in ultrapure water, which increased the negative charge of MLG/CNC. In general, MLG/CNC also showed a trend; that is, the higher concentration of the CNC aqueous suspension, the lower the zeta potential of MLG/CNC; the greater the amount of MLG added, the lower the zeta potential of MLG/CNC. As mentioned above, samples 7–12 were diluted with ultrapure water. When the dispersant was NMP and alcohol, the higher the amount of MLG, the higher the zeta potential, which was contrary to the trend of CNC aqueous suspension as the dispersant. Generally, the NMP has a similar surface energy to graphene, which could reduce the enthalpy and promote the stabilization of MLG/NMP suspension (Yang et al., 2020). Analysis of zeta results showed that CNC aqueous suspension was stabilized by the repulsion of negative charges on the particle surface, while MLG was also negatively charged. The two repel each other, making the MLG/CNC aqueous suspension stable without the sedimentation of MLG.

### Sedimentation Analysis

**FIGURE 4:**
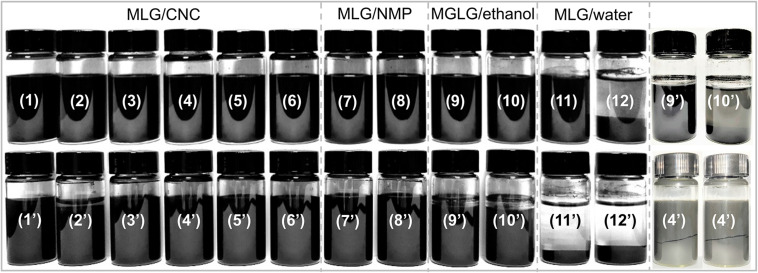
Digital photographs of each sample as prepared (1–12) and 135 days later (1’–12’).

As shown in Figure 4, sample 12 (1.0 wt% MLG dispersed in ultrapure water) was the only sample that has sedimented at the beginning of its preparation, and according to the above description, its zeta potential reached −29.1 mV. After 135 days of sedimentation experiment, most MLG/CNC (sample 1, 3–6) and all the MLG/NMP showed no visible sedimentation. MLG precipitation occurred in sample 2 (MLG/CNC), sample 10 (MLG/ethanol), and all the MLG/ultrapure water samples. Besides, sample 4 did not precipitate, but it was divided into two parts. This phenomenon was not found in other samples.

After 135 days, some of the alcohol in samples 9 and 10 (MLG/ethanol) evaporated, leaving a few circles of black MLG on the inner wall of the sample bottles. This result showed that CNC at an appropriate concentration can be used as an adjuvant to improve the dispersion ability of MLG in aqueous suspension.

### AFM and TEM Analysis

**FIGURE 5:**
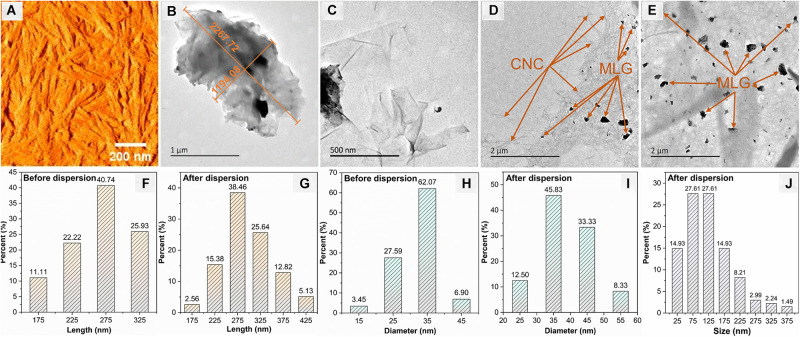
The morphologies of CNC and MLG/CNC. **(A)** AFM image of CNC. **(B,C)** TEM images of non-ball-milled MLG at different magnifications. **(D,E)** TEM images of MLG/CNC. **(F,G)** The length distribution of CNC before and after dispersion. **(H,I)** The diameter distribution of CNC before and after dispersion. **(J)** The size distribution of MLG after dispersion.

The AFM image (Figure 5A) shows the morphology of the CNC with a typical rod-like structure, and Figures 5F–I show the length and diameter (measured by Adobe Photoshop CC 2015) distribution of CNC before and after dispersion. The size of CNC has not changed significantly before and after dispersion; the length was concentrated at 275 nm, and the largest proportion of diameter was 35 nm. The TEM images (Figures 5B,C) indicated that the size of non-ball-milled MLG was up to 2 μm or more. It can be known from Figure 5J that the size of the MLG dispersed in CNC aqueous suspension was concentrated in 50–150 nm, which was smaller than the particle size after ball-milling. This was because after dispersing the ball-milled MLG into the CNC aqueous suspension, sonicating it for 10 min will further reduce the size of the MLG. Figure 5C shows that relatively thin MLG has a wrinkled translucent structure. According to Zhu et al., the optical transmittance of single-layer graphene was almost 97% (Zhu et al., 2014), and the more layers, the lower the light transmittance. Wrinkles on the surface of graphene were not defects; they were a common phenomenon in two-dimensional membranes (Zhu et al., 2012). Wrinkles can be generated whether the graphene was peeled from three-dimensional highly oriented pyrolytic graphite to two-dimensional graphene or grown on the surface (Liu et al., 2011). Figures 5D,E show that countless CNCs have formed a “3D net” with MLG attached. The size distribution of CNC and MLG before and after dispersion showed that neither CNC nor MLG was aggregated after dispersion, and CNC can be used as an adjuvant to disperse MLG in an aqueous suspension.

### Absorbance Analysis

**FIGURE 6:**
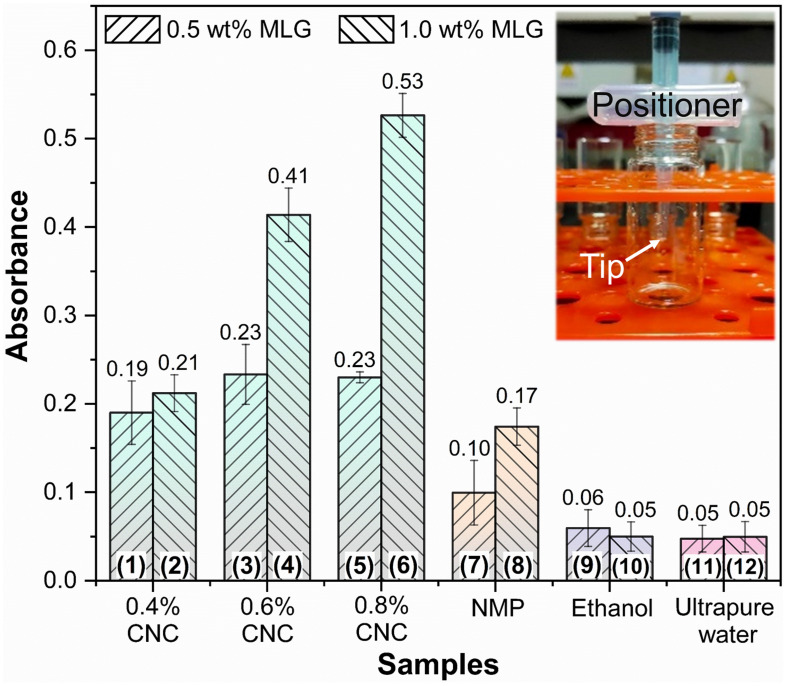
The absorbance bar plot at 660 nm of samples. The inset represents the “positioner,” which ensures that samples of the same height were tested in the bottles.

The absorbance at 660 nm is shown in Figure 6; the results of different adjuvants were significantly different. For the same adjuvant or dispersant (including the same concentration of CNC), except for MLG/ethanol, the absorbance of the sample containing 1.0 wt% MLG was higher than 0.5 wt%. Among them, the most significant difference was between sample 6 and sample 5. The absorbance of sample 6 was 2.3 times than that of sample 5. The smallest difference was between sample 11 and sample 12, which were almost the same. For the samples of CNC as adjuvant, when the addition of MLG was 0.5 wt%, as the CNC concentration increased, the absorbance first increased and then remained unchanged. When the amount of MLG was 1.0 wt%, the absorbance increased with an increase of CNC concentration. In particular, when ethanol was used as dispersant, the absorbance of sample 10 (with 1.0 wt% MLG) was lower than that of sample 9 (with 0.5 wt% MLG). According to Lambert–Beer law, the higher the absorbance, the higher the concentration of MLG dispersion (Zhang et al., 2013), and the better the dispersion effect of the dispersant on MLG.

The absorbance of samples 9–12 was very close, but in Figure 4, it can be observed that the MLG in samples 11 and 12 has been significantly precipitated, and the upper liquid has become transparent. The color of samples 9 and 10 was black; after adjusting the brightness of the digital photo, precipitation can only be observed in sample 10. This showed that the results obtained by visual observation were not accurate in this study.

### FTIR Analysis

**FIGURE 7:**
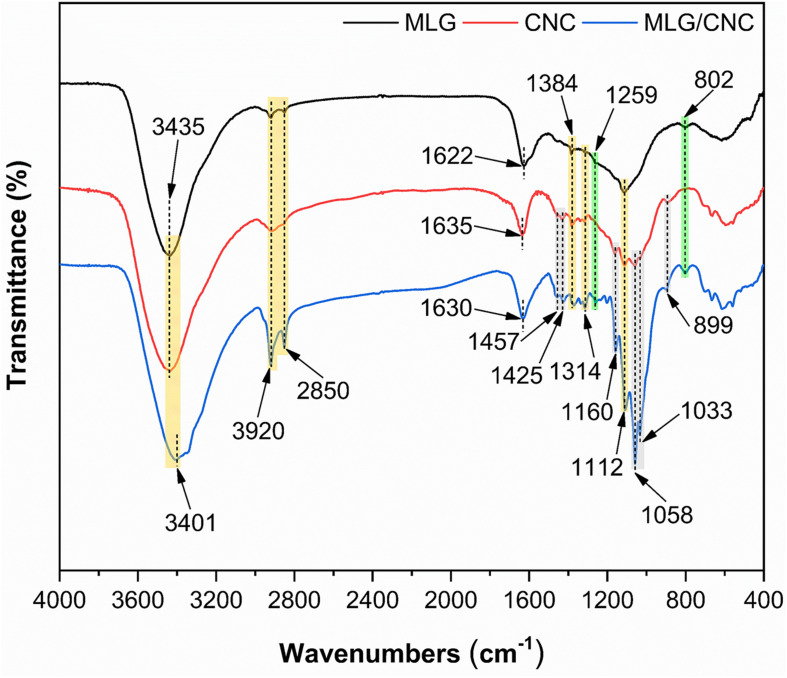
The FTIR spectroscopy of dried MLG, CNC, and MLG/CNC.

The FTIR spectra (Figure 7) of dried MLG and CNC were further compared with dried MLG/CNC. The peaks at 3,435 and 3,401 cm^–1^ were assigned to O–H stretching vibration. The peaks at 3,920, 2,850, and 1,384 cm^–1^ were caused by the symmetrical stretching vibration, asymmetric stretching vibration, and bending vibration of C–H (Jin et al., 2016), respectively. The peaks of 1,425, 1,160, 1,112, 1,058, and 899 cm^–1^ were observed, which were associated with the typical feature groups of cellulose (Liu et al., 2017). The green area peaks showed obvious appearance on the spectra of MLG and MLG/CNC but were not obvious or did not appear on the CNC spectrum. The peaks at 1,259 and 802 cm^–1^ indicated the C–H deformation (Leng et al., 2017). Correspondingly, the peaks in the gray area indicate that they appear on the spectra of CNC and MLG/CNC, but were not obvious on the MLG spectrum. The spectrum peaks at 1,457 and 1,033 cm^–1^ represented the −CH_2_ symmetric bending vibration and C–O stretching vibration (Wu et al., 2019a), respectively. The characteristic peak at 1,622 cm^–1^ of MLG corresponded to C–C (aromatic ring) stretching vibration (Xiong et al., 2016). Other peaks at 1,635 cm^–1^ of CNC and 1,630 cm^–1^ of MLG/CNC belonged to the moisture that remained in the samples.

This result showed that the FTIR spectrum of MLG/CNC has special peaks in both the MLG and CNC spectrum, which showed that the two were closely integrated. This result also verified the conclusion of TEM analysis. In addition, the vibration peaks of C–H and O–H that appeared in MLG indicated that the MLG used in this study was not purified.

### Raman Spectroscopy

**FIGURE 8:**
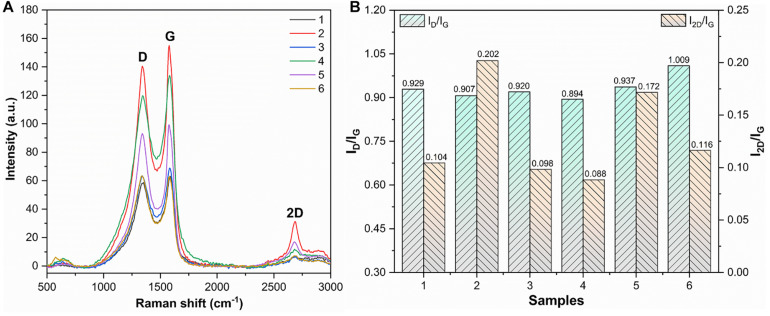
Raman spectra, I_D_/I_G_, and I_2D_/I_G_ of dried MLG/CNC. **(A)** The Raman spectra of dried MLG/CNC. **(B)** The I_D_/I_G_ and I_2D_/I_G_ bar plot of Raman spectra of dried MLG/CNC.

Raman spectra were used to evaluate the structure and defects of MLG. As shown in Figure 8A, the MLG/CNC has the typical Raman characteristic peaks. One was the D peak located near 1,350 cm^–1^, and the other was the G peak located near 1,580 cm^–1^ and the 2D peak around 2,670 cm^–1^ (Dresselhaus et al., 2010). In general, the D-band intensity represents the degree of disordered carbon in the composite and the intensity of the G-band represents the number of sp2 hybridizations. The larger intensity ratio I_D_/I_G_ means larger disorder and defect. The 2D band originated from a two-phonon double-resonance process, which was closely associated with the band structure of graphene layers; in single-layer graphene, I_2D_ is nearly twice as strong as I_G_ (Hu et al., 2019). If the number of graphene layers does not exceed five layers, the number of graphene layers can be determined directly based on the ratio of I_2D_/I_G_ (Liu et al., 2013). The I_D_/I_G_ and I_2D_/I_G_ are shown in Figure 8B.

It was clearly known that the smaller the ratio of I_2D_/I_G_, that is, the fewer layers. Figure 8B shows that sample 4 (1.0 wt% MLG–0.6% CNC) has the smallest I_D_/I_G_ value of 0.894; that is, it has the lowest defects and disorder. Sample 2 has the largest I_2D_/I_G_ value (0.202), which was the minimum number of layers in the MLG/CNC. Figure 8B also shows that sample 4 has the smallest values of I_2D_/I_G_ (0.088) and I_D_/I_G_ (0.894) at the same time; that is, it has the lowest defects and disorder, but also has the largest number of layers, and graphene aggregation may have occurred. The highest I_2D_/I_G_ value belonged to sample 2 (1.0 wt% MLG–0.4% CNC), and its I_D_/I_G_ value was the second smallest (0.907), which can be used as the most preferred concentration and addition ratio among the MLG/CNC samples.

## Conclusion

We demonstrated a simple, eco-friendly approach to improve the dispersion ability of MLG in aqueous suspension. After ball-milling, the MLG was dispersed into CNC aqueous suspensions. In addition, NMP, ethanol, and ultrapure water were used as control groups. By observing the sedimentation experiment results of MLG/CNC and other control groups after storage of 135 days, as well as the characterization of zeta potential, AFM, TEM, absorbance, FTIR spectroscopy, and Raman spectroscopy, the effect of CNC as adjuvant to improve the MLG dispersion ability in aqueous suspension was studied. The zeta potential and FTIR results show that the stability of MLG/CNC meets the requirement and the combination of CNC and ball-milled MLG is stable in aqueous suspension. Results from AFM and TEM show that the size of CNC did not change after dispersion, but the size of MLG became smaller. The highest concentration of the samples is obtained by dispersing 1.0 wt% MLG into 0.8% CNC aqueous suspension. When the CNC concentration is 0.4% and the addition amount of MLG is 1.0 wt%, the MLG has the least defects and the thinnest thickness, which can be used as the best experimental parameters. Overall, CNC plays an obvious role in helping MLG disperse in aqueous suspension, while MLG does not agglomerate, which can provide a solution to the problem of MLG dispersion in aqueous suspension.

## Data Availability Statement

The original contributions presented in the study are included in the article/supplementary material, further inquiries can be directed to the corresponding author/s.

## Author Contributions

HZ, YW, and JZ: conceptualization. HZ and YW: writing—original draft preparation. HD, YB, and FY: investigation. HZ, HJ, and XX: methodology. HJ, XX, and YW: funding acquisition. All authors have read and agreed to the published version of the manuscript.

## Conflict of Interest

HJ and XX were employed by the company Dehua Tubao New Decoration Material Co., Ltd., Huzhou, China. The remaining authors declare that the research was conducted in the absence of any commercial or financial relationships that could be construed as a potential conflict of interest.
